# A multisite longitudinal evaluation of patient characteristics associated with a poor response to non-surgical multidisciplinary management of low back pain in an advanced practice physiotherapist-led tertiary service

**DOI:** 10.1186/s12891-020-03839-5

**Published:** 2020-12-03

**Authors:** Shaun O’Leary, Maree Raymer, Peter Window, Patrick Swete Kelly, Darryl Lee, Linda Garsden, Rebecca Tweedy, Ben Phillips, Will O’Sullivan, Anneke Wake, Alison Smith, Sheryl Pahor, Luen Pearce, Rod McLean, David Thompson, Erica Williams, Damien Nolan, Jody Anning, Ian Seels, Daniel Wickins, Darryn Marks, Brendan Diplock, Vicki Parravicini, Linda Parnwell, Bill Vicenzino, Tracy Comans, Michelle Cottrell, Asaduzzaman Khan, Steven McPhail

**Affiliations:** 1 School of Health and Rehabilitation Sciences, University of Queensland, Brisbane, Australia; 2 Physiotherapy Department, Royal Brisbane and Women’s Hospital, Brisbane, Australia; 3 Physiotherapy Department, Townsville Hospital, Townsville, Australia; 4 Physiotherapy Department, Cairns Hospital, Cairns, Australia; 5 Physiotherapy Department, Princess Alexandra Hospital, Brisbane, Australia; 6 Physiotherapy Department, Nambour Hospital, Nambour, Australia; 7 Physiotherapy Department, Redcliffe Hospital, Redcliffe, Australia; 8 Physiotherapy Department, Gold Coast Hospital, Gold Coast, Australia; 9 Physiotherapy, Faculty of Health Sciences & Medicine, Bond Institute of Health and Sport, Bond University, Robina, Australia; 10 Physiotherapy Department, Logan Hospital, Logan, Australia; 11 Centre for Health Services Research, University of Queensland, Brisbane, Australia; 12 Clinical Informatics Directorate, Metro South Hospital and Health Service, Brisbane, Australia; 13 Australian Centre for Health Services Innovation & Centre for Healthcare Transformation, School of Public Health & Social Work, Queensland University of Technology, Brisbane, Australia

**Keywords:** Low back pain, Tertiary care, Risk factors, Outcome, Non-surgical management

## Abstract

**Background:**

Non-surgical multidisciplinary management is often the first pathway of care for patients with chronic low back pain (LBP). This study explores if patient characteristics recorded at the initial service examination have an association with a poor response to this pathway of care in an advanced practice physiotherapist-led tertiary service.

**Methods:**

Two hundred and forty nine patients undergoing non-surgical multidisciplinary management for their LBP across 8 tertiary public hospitals in Queensland, Australia participated in this prospective longitudinal study. Generalised linear models (logistic family) examined the relationship between patient characteristics and a poor response at 6 months follow-up using a Global Rating of Change measure.

**Results:**

Overall 79 of the 178 (44%) patients completing the Global Rating of Change measure (28.5% loss to follow-up) reported a poor outcome. Patient characteristics retained in the final model associated with a poor response included lower Formal Education Level (ie did not complete school) (Odds Ratio (OR (95% confidence interval)) (2.67 (1.17–6.09), *p* = 0.02) and higher self-reported back disability (measured with the Oswestry Disability Index) (OR 1.33 (1.01–1.77) per 10/100 point score increase, *p* = 0.046).

**Conclusions:**

A low level of formal education and high level of self-reported back disability may be associated with a poor response to non-surgical multidisciplinary management of LBP in tertiary care. Patients with these characteristics may need greater assistance with regard to their comprehension of health information, and judicious monitoring of their response to facilitate timely alternative care if no benefits are attained.

## Background

Low back pain (LBP) accounts for the greatest number of years lived with disability of all health conditions [1]. Many patients with chronic disabling LBP are referred to tertiary hospitals for specialist opinion regarding management which can be a substantial load on hospital services [2]. Multidisciplinary non-surgical management is usually the first line of care for these patients as surgical outcomes are similar but have greater potential for adverse events [3]. It is suggested that non-surgical multidisciplinary management of LBP is aligned to the biopsychosocial conceptualization of chronic pain [3, 4] delivered by a team of multidisciplinary health professionals providing a combination of coordinated educational, physical and psychological interventions [3]. While systematic review and meta-analysis evidence supports the benefits of non-surgical multidisciplinary management for LBP in the long term, not all patients benefit and treatment effects may be modest [3, 5]. Obvious advantages exist in the early identification of patient characteristics that may be associated with a poor response to non-surgical multidisciplinary management. Firstly this knowledge may facilitate a more tailored management approach for patients with these identified characteristics, and secondly, their response to management may be more closely monitored to facilitate timely referral if no benefits are observed.

The identification of patient characteristics associated with an intervention response needs to be specific to the patient population, health service setting, and intervention type, of interest. Extrapolating findings to patients with LBP regarding their likely response to an intervention is potentially misleading if based on studies conducted in a different health setting (eg. primary versus tertiary), where marked variation in patient demographics, level of disability, and intervention content may exist [6]. There is limited literature reporting patient characteristics associated with a poor response to non-surgical multidisciplinary management of LBP specifically delivered in tertiary care settings. Studies in LBP have tended to explore risk factors with regards to the transition to chronicity [7–9], delayed recovery [10], and treatment response to single or combined interventions (eg. acupuncture, manipulation, exercise, cognitive behavioural therapy) [11].

We previously published a retrospective audit of medical records investigating patient characteristics associated with a poor response to non-surgical multidisciplinary management of chronic LBP [6]. The audit was conducted within an advanced practice physiotherapist-led multidisciplinary orthopaedic service (referred herein as the ‘service’) embedded in multiple tertiary hospital facilities. Patients had been referred to these tertiary hospitals for medical specialist opinion but on initial triage were deemed appropriate for a trial of non-surgical multidisciplinary management within the service. The service at each facility employs experienced musculoskeletal physiotherapists (service leader) with appropriate postgraduate qualifications (eg. Masters in Musculoskeletal Physiotherapy) who assesses the patient and coordinates their care [2]. The non-surgical management is patient-centred and multidisciplinary conducted by registered health professionals (as required; physiotherapy, occupational therapy, dietetics, and/or psychology) with an emphasis on progression from supported to non-supported self-management to pragmatically address the mix of biopsychosocial factors potentially underlying LBP [3, 4]. Our audit showed that patients managed within the service for LBP who had low pain self-efficacy and coexisting cervical or thorax pain at the initial consultation were at greater odds of experiencing a poor response to management [6]. However, there were marked limitations of this study due its retrospective nature and limited patient characteristics evaluated (demographic, general health, quality of life, psychological, symptom characteristics, functional level, prior management, radiological findings) largely due to a lack of standardised recording within medical records [6].

The purpose of this prospective longitudinal study was to further explore patient characteristics associated with a poor response to non-surgical management of LBP specific to the same advanced practice physiotherapist-led multidisciplinary orthopaedic service. In particular this prospective study was able to incorporate a broader selection of patient characteristics within each category (demographic and social, general and global health, psychological, condition specific signs and symptoms (including physical and radiological)) using standardised recording compared to the previous study [6].

## Methods

### Study design

This prospective longitudinal study was conducted across 8 tertiary public hospital facilities. The study explored the relationship between patient characteristics recorded at the initial examination conducted by the service leader (21 service leaders across eight facilities) and a poor response to non-surgical multidisciplinary management of LBP. The approach to identify patient characteristics associated with a poor response to management as a clinical priority is consistent with our previous study [6]. Multisite ethical approval was received from the Institutional Medical Research Ethics Committee (HREC/13/QRBW/80) for the project as well as site specific approval from each of the participating hospitals. This manuscript follows STROBE reporting guidelines [12].

### Participants

All participants were recruited within the service at participating hospitals between January 2014 to December 2016 with follow-up completed in July 2017. Potential participants had been triaged from specialist neurosurgical and orthopaedic outpatient waiting lists by the medical consultant and/or service leader to undergo non-surgical management of their LBP within the service. At the initial examination the service leader further screened the participant for study eligibility. Participants were included based on reported LBP which could include pain, muscle tension or stiffness localised below the costal margin and above the inferior gluteal folds, with or without leg pain [13]. Participants were excluded if they presented with a potentially serious medical condition (red flags), signs of central nervous system involvement such as cord or cauda equina signs (eg. positive Babinski or clonus), an active inflammatory condition (e.g. Ankylosing spondylitis, Rheumatoid Arthritis), current fractures, back pain of hip origin, severe symptoms likely to be aggravated by therapies, significant or unstable neurovascular involvement, or if the referring medical practitioner or patient specifically requested specialist medical consultation.

### Sample size estimation

It was estimated that 224 cases should be the target sample size for analysis. This was based on an expected 50% non-responder rate [6], and assumed i) the inclusion of up to 7 explanatory variables in a final multivariable (logistic family) model and ii) *n* = 16 poor responders per variable, which may be considered more conservative than required based on prior simulations [14]. To account for up to 15% drop out, it was estimated that a minimum target of 264 participants were to be recruited for this study.

### Criteria for a poor response to outcome (dependent variable)

Patients rated their overall perception of change since beginning treatment on a 15-point Global Rating of Change (GROC) scale [15–17]. Specifically patients were asked “With respect to your low back condition, how would you describe yourself now compared to 6 months ago when you entered the study?” Responses were dichotomised as either poor (scores between − 7 (A very great deal worse) and + 1 (Almost the same, hardly any better at all)) or positive (scores between + 2 (A little better) and + 7 (a very great deal better)) to account for the generally high severity of LBP presentations seen within this speciality tertiary service. Specifically scores between − 7 and + 1 represented worsening or negligible change in the disorder, and therefore were considered a poor response. The GROC was assessed at 6 months following entry to the service consistent with our previous study, considered as a conservative timeframe to evaluate impact of management in this service [6].

### Potential explanatory variables (independent variables)

Patient characteristics evaluated for their association with outcome were recorded at the initial consultation with the service leader. Service leaders were engaged in the selection of these variables as they were obtained from routine demographic, social and clinical information from the patient interview, self-report questionnaires capturing condition specific as well as general health and psychological information, as well as information gained from standardised physical tests recorded by the service leader who conducted physical assessments, and reviewed radiological imaging findings. Selection of measures was therefore pragmatic to incorporate measures already collected and considered important by the service using instruments (eg. questionnaires, goniometers) accessible across all service facilities, while also ensuring relevant information was captured within the biopsychosocial context of LBP [3, 4]. They were classified under one of four categories outlined below (as listed in Table 1) and include:

1. *Demographic and social measures including –* Age (years), gender (male/female), english first language (yes/no), education level (yes/no; school incomplete, completed secondary school, completed Technical and Further Education (TAFE)/Trade/ University), work status (yes/no; employed (full or part-time), unemployed, retired), marital status (yes/no; married/defacto, single), dependents (yes/no).
2. *General and global health measures including –* Body Mass Index (kilogram/meters^2^), disability benefits (yes/no), smoking status (yes/no), comorbidities (number/18 listed conditions), total body pain areas (number/18 segmented body chart), health related quality of life (utility score/1, higher scores indicate higher quality of life) measured with the Assessment of Quality of Life questionnaire (AQoL – 6D) that has acceptable validity and reliability [18].
3. *Psychological measures included –* General psychological distress was evaluated with the Depression, Anxiety and Stress Scale (DASS-21) with each of the three dimensions of depression (score/42), anxiety (score/42), and stress (score/42) scored (higher scores indicate higher psychological distress) separately, shown to have favourable measurement reliability and construct validity [19]. Pain related psychological measures included the Pain Self-Efficacy Questionnaire (score/60, higher scores indicate greater self-efficacy) assessing the participants’ confidence in performing activities (eg. household chores, socialising, work) despite their pain [20] which has acceptable reliability and validity [21], and the Örebro Musculoskeletal Pain Screening Questionnaire (ÖMPSQ - short) used to identify yellow flags (eg. distress, fear avoidance beliefs) and risk of pain related work disability (score/100, higher scores indicate a more severe presentation) with acceptable reliability and validity [22, 23]. A measure of the patient’s expectations of benefit (score/10, higher scores indicate higher expectations of benefit) from the non-surgical management approach was also taken using a visual analogue scale anchored by the terms “No benefit” and “Extreme benefit”.
4. *Condition specific symptoms and signs included –* The patients’ self-reported level of back disability (score/100, higher scores indicate more severe disability) was evaluated with the Oswestry Disability Index (ODI) which has been shown to have acceptable reliability and validity [24]. This index is used to quantify disability for low back pain and contains items concerning pain intensity and function (eg. lifting, self-care) [25]. Participant nominated functional deficits were evaluated with the Patient Specific Functional Scale (score/10, higher scores indicate better function) which has acceptable reliability and validity in low back pain conditions [26, 27]. The potential presence of neuropathic pain was evaluated with the Leeds Assessment of Neuropathic Symptoms and Signs (S-LANSS) pain scale shown to be valid and reliable self-report instrument for indicating the potential presence of predominantly neuropathic pain (yes/no, score of ≥12 points) [28]. Other self-reported information regarding the condition included; symptom duration (yes/no; > 12 months), traumatic onset (yes/no), previous orthopaedic surgery for the same condition (yes/no), and symptom distribution including coexisting thorax and/or cervical spine pain (yes/no), bilateral (as opposed to unilateral) low back pain (yes/no), and the presence of leg pain (yes/no) and or leg anaesthesia/paraesthesia (yes/no).

**Table 1:** Means (± standard deviation) (normally distributed continuous data), medians (IQR) (non-normally distributed continuous data) and row participant percentages (n) (categorical data) for the independent variables grouped for participants dichotomised as a poor response (GROC ≤ + 1) or a positive response (GROC ≥ + 2) to the non-surgical multidisciplinary management of LBP. OR’s reflect either an increased (OR > 1) or decreased (OR < 1) odds of a poor response associated with higher scores (continuous measures expressed as a 1 unit increase in measure) or presence of patient characteristics (categorical measures)

Variables	Poor Response (***n*** = 79)	Positive Response (***n*** = 99)	Unadjusted OR (95% Confidence Intervals)	***P***-Value
**Demographic and Social Measures**				
Age (years)	53.61 ± 14.39	53.74 ± 16	1 (0.98–1.02)	0.96
Gender (% male (n))	50.5% (48)	49.5% (47)	1.71 (0.94–3.12)	0.08*
English First Language (% yes (n))	43.5% (70)	56.5% (91)	0.68 (0.25–1.86)	0.46
Highest Level of Education				
Completed Tafe/Trade/ University (% yes (n))	30% (23)	70% (53)	Referent	
Completed Secondary School (% yes (n))	53% (16)	47% (14)	2.63 (1.11–6.28)	0.03*
School Incomplete (% yes (n))	58% (37)	42% (27)	3.16 (1.58–6.34)	0.001*
Work Status				
Employed (% yes (n))	36% (29)	64% (52)	Referent	
Unemployed (% yes (n))	58% (14)	42% (10)	2.51 (0.99–6.36)	0.05*
Retired (% yes (n))	45% (26)	55% (32)	1.46 (0.73–2.9)	0.28
Married/Defacto Relationship (% yes (n))	43% (44)	57% (59)	0.88 (0.48–1.6)	0.67
Dependents (% yes (n))	43% (21)	57% (28)	0.92 (0.47–1.78)	0.8
**General and Global Health Measures**				
Body Mass Index (kilogram/meters^2^)	30.61 ± 7.78	30.52 ± 7.11	1 (0.96–1.05)	0.94
Disability Benefits (% yes (n))	66% (21)	34% (11)	2.95 (1.32–6.57)	0.008*
Smoking Status (% yes (n))	56% (27)	44% (21)	1.9 (0.97–3.72)	0.06*
Comorbidities (number)	2 (1–3)	1 (1–2)	1.03 (0.87–1.23)	0.72
Total Body Pain Areas (number/18 body regions)	5 (3–8)	5 (3–7)	1.02 (0.94–1.12)	0.64
Quality of Life (utility score/1)	0.54 (0.4–0.68)	0.65 (0.49–0.79)	0.11 (0.02–0.54)	0.007*
**Psychological Measures**				
DASS-21				
Depression Score (score/42)	10 (4–24)	8 (4–18)	1.02 (0.99–1.05)	0.14
Anxiety Score (score/42)	8 (3–15)	6 (0–12)	1.02 (0.99–1.06)	0.23
Stress Score (score/42)	12 (4–25)	12 (5–18)	1.02 (0.99–1.05)	0.23
Pain Self-Efficacy Questionnaire (score/60)	27.26 ± 13.65	32.2 ± 13.84	0.97 (0.95–1)	0.03*
ÖMPSQ (score/100)	62.99 ± 14.31	55.16 ± 14.91	1.04 (1.02–1.06)	0.001*
Patient Expectations of Benefit (score/10)	5.8 ± 2.58	6.26 ± 2.78	0.94 (0.84–1.05)	0.27
**Condition Specific Symptoms and Signs**				
Oswestry Disability Index (score/100)	46.1 ± 15.69	37.43 ± 15.16	1.04 (1.01–1.06)	0.001*
Patient Specific Functional Scale (score/10)	4.03 ± 1.77	4.29 ± 1.77	0.92 (0.76–1.11)	0.39
S-LANSS Pain Score (% ≥ 12 points (n))	49% (35)	51% (37)	1.35 (0.74–2.48)	0.33
Symptom Duration (% > 12 months (n))	49% (66)	51% (68)	2.35 (1.1–4.98)	0.03*
Traumatic Onset (% yes (n))	49% (31)	51% (33)	1.44 (0.77–2.71)	0.25
Previous Surgery (% yes (n))	47% (7)	53% (8)	1.1 (0.38–3.18)	0.86
Symptom Distribution				
Thorax and/or Cervical Spine Pain (% yes (n))	46% (41)	54% (48)	1.15 (0.63–2.07)	0.65
Bilateral Low Back Pain (% yes (n))	48% (57)	52% (62)	1.65 (0.86–3.18)	0.13
Leg Pain (% yes (n))	45% (58)	55% (71)	1.16 (0.59–2.3)	0.67
Leg Paraesthesia/Anaesthesia (% yes (n))	49% (40)	51% (41)	1.5 (0.82–2.74)	0.18
Physical Findings				
Lumbar Extension Range (°)	18.92 ± 12.12	20.55 ± 10.91	0.99 (0.96–1.01)	0.36
Lumbar Flexion Range (°)	63.3 ± 26.33	68.52 ± 23.26	0.99 (0.98–1)	0.17
Nerve Conduction (% positive pattern (n))	38% (16)	62% (26)	0.71 (0.34–1.48)	0.37
Nerve Mechanosensitivity (% yes (n))	42% (36)	58% (49)	0.92 (0.51–1.68)	0.79
Radiological Findings (all OR’s calculated relative to reference of no abnormality reported on imaging)				
Lumbar Single Level Degenerative Change				
Absent (% yes (n))	43% (55)	57% (72)	Referent	
Mild (% yes (n))	62% (8)	38% (5)	2.09 (0.65–6.76)	0.22
Moderate (% yes (n))	32% (6)	68% (13)	0.6 (0.22–1.69)	0.34
Severe (% yes (n))	46% (5)	54% (6)	1.09 (0.32–3.76)	0.89
Lumbar Multilevel Degenerative Change				
Absent (% yes (n))	47% (24)	53% (27)	Referent	
Mild (% yes (n))	43% (12)	57% (16)	0.84 (0.33–2.14)	0.72
Moderate (% yes (n))	40% (26)	60% (39)	0.75 (0.36–1.57)	0.45
Severe (% yes (n))	48% (13)	52% (14)	1.04 (0.41–2.66)	0.93
Central Canal Stenosis				
Absent (% yes (n))	45% (53)	55% (65)	Referent	
Mild (% yes (n))	38% (8)	62% (13)	0.75 (0.29–1.96)	0.56
Moderate (% yes (n))	48% (9)	52% (10)	1.1 (0.42–2.91)	0.84
Severe (% yes (n))	39% (5)	61% (8)	0.77 (0.24–2.48)	0.66
Foraminal Stenosis				
Absent (% yes (n))	39% (43)	61% (68)	Referent	
Mild (% yes (n))	46% (6)	54% (7)	1.36 (0.43–4.30)	0.61
Moderate (% yes (n))	53% (17)	47% (15)	1.79 (0.81–3.96)	0.15
Severe (% yes (n))	60% (9)	40% (6)	2.37 (0.79–7.14)	0.12
Lateral Stenosis				
Absent (% yes (n))	44% (56)	56% (70)	Referent	
Mild (% yes (n))	40% (6)	60% (9)	0.83 (0.28–2.48)	0.74
Moderate (% yes (n))	38% (8)	62% (13)	0.77 (0.3–1.99)	0.59
Severe (% yes (n))	56% (5)	44% (4)	1.56 (0.4–6.09)	0.52
Disc Pathology (% yes (n))	42% (57)	58% (79)	0.68 (0.32–1.44)	0.31
Vertebral Body Pathology (% yes (n))	59% (13)	41% (9)	2.03 (0.82–5.04)	0.13
Zygopophyseal Joint Pathology (% yes (n))	42% (39)	58% (54)	0.84 (0.46–1.55)	0.58
Spondylolysis (% yes (n))	41% (7)	59% (10)	0.88 (0.32–2.42)	0.8
Spondylolisthesis (% yes (n))	55% (22)	45% (18)	1.8 (0.88–3.67)	0.11
Nerve Comprise (% yes (n))	39% (37)	61% (59)	0.61 (0.33–1.13)	0.11
Radiology Findings - Poor Prognosis (% yes (n))	44% (24)	56% (30)	1.04 (0.54–2.01)	0.9

*denotes variables with a univariate relationship (*p* ≤ 0.1) with a poor response in outcome that were considered for the final analysis. *DASS-21* Depression, Anxiety and Stress Scale, *ÖMPSQ – short* Örebro Musculoskeletal Pain Screening Questionnaire, *S-LANSS* Leeds Assessment of Neuropathic Symptoms and Signs

Physical examination measures conducted by the advanced musculoskeletal physiotherapist were also recorded. Lumbar spine mobility included lumbar flexion and extension range of motion (°) measured with an inclinometer [29, 30]. Clinical assessment of lower limb nerve conduction (reflexes, muscle power, light touch sensation) was undertaken and clinicians were asked to identify if, in their clinical judgement, the test results were consistent with a recognised pathoanatomical (radicular) pattern of nerve compromise (yes/no). A measure of nerve mechanosensitivity was also recorded during a straight leg raise (both lower limbs evaluated). The test was rated as positive (yes/no) if symptoms evoked by the straight leg raise could be altered with sensitisation manoeuvres remote to the area of pain (ie structural differentiation) [31–33] which has been shown to have moderate to substantial inter-rater agreement (kappa 0.49–0.8) [34, 35].

Available radiological reports (X-Ray, MRI, CT) or radiological findings documented in the medical notes by a medical consultant, were reviewed and relevant findings recorded regarding; the presence and severity (absent, mild, moderate, severe) of lumbar single or multilevel degenerative change, central canal stenosis, foraminal or lateral stenosis. Recordings were also made as to the reported presence (yes/no) of; disc pathology, vertebral body pathology, zygopophyseal joint pathology, spondylolysis, spondylolisthesis, or nerve comprise. Clinicians were also asked to judge if they considered the radiological findings to be suggestive of a poor prognosis (yes/no). Relevance of the imaging with regard to how recently it was taken was at the service leader’s discretion.

### Procedure

Following screening with the service leader eligible participants were invited to participate and provided informed consent. Consented participants then completed an initial examination during which all explanatory variable measures were collated. This included collection of forms containing demographic information as well as the self-report questionnaires (that had been pre-mailed and completed by patients prior to the initial session as is the standard procedure in these clinics), as well as the relevant measures recorded during the patient interview and physical examination. To ensure measures were collated in a standardised manner all service leaders collecting measures across sites were provided with a training manual and a training session before the commencement of the study. Additional training was provided to any clinical leaders if requested. The collection of baseline variables was facilitated by a designated study research officer who worked in conjunction with the services at each site.

Participants then underwent non-surgical patient-centred multidisciplinary (as required; physiotherapy, occupational therapy, dietetics, and/or psychology) management within the service at the respective facilities. Each patient’s management (duration of management period, disciplines consulted, interventions (eg. education/advice, lifestyle modification, dietary modifications, exercise prescription, manual therapy) was pragmatically based on the initial examination findings of the clinical leader and the clinical discretion of the involved discipline-specific treatment providers who were all registered health professionals. At 6 months following entry to the study, the research officer mailed the GROC measure to the participants, with further contact made by phone (2–3 attempts) as necessary to ensure the outcomes were returned. This 6 month follow up measure was performed independently of the clinical service at all sites. Participants may or may not have been still receiving management within the service at this time point, which was at the discretion of the respective services.

### Statistical analysis

Analyses were conducted using Stata 13 (StataCorp. 2013. Stata Statistical Software: Release 13. College Station, TX: StataCorp LP.). Participant characteristics and outcomes were described using conventional descriptive statistics with mean (SD) for normally distributed data, median (IQR) for non-normally distributed data, and number (%) for categorical data. Generalised linear models with binomial family and logit link with site as a random effect were used to examine the association between potential explanatory variables, with patients being classified as either having a poor response (GROC score ≤ + 1) or positive response (GROC score + 2 − + 7) to multi-disciplinary non-surgical management in this sample. To select the most appropriate explanatory variables to carry forward to a multivariable model, univariate analyses were initially conducted and an unadjusted Odds Ratio (95% confidence interval) was calculated for each variable. To identify the most parsimonious selection of explanatory variables associated with a poor response, only explanatory variables that displayed a univariate relationship of *p* ≤ 0.1 with the reference score (GROC score ≤ + 1) were considered for the multivariable analyses [36]. Eligible variables were then further screened as to their relative level of potential clinical impact and priority to take to the final model. This was particularly the case if multiple variables from a similar domain (eg. psychological measures, physical measures) were eligible. Potential multi-collinearity issues between eligible explanatory variables were then evaluated with a Spearman’s rho (r_s_) correlational coefficient as it is appropriate for both continuous and discrete ordinal variables [37]. If variables were shown to have significant moderate (r_s_ = 0.4–0.6) or strong (r_s_ = 0.7–0.9) correlations [38, 39], only one variable was selected (investigator’s choice based on clinical reasoning) to be carried forward for the final model. After accounting for potential non-independence associated with site by including site as a random effect, and the aforementioned variable selection process to avoid multi-collinearity, we did not detect any breaches of model assumptions in the final model. The c-statistic was also calculated for the final model. Additionally in the final model findings for the ODI were expressed as per 10 points (of the 100 point scale) for ease of interpretation. To ensure findings were robust against this variable selection process which included investigator judgement, any correlated variables that were not selected were substituted into the multivariable analysis in place of the selected variable as sensitivity analyses to determine if findings were impacted by the choice of included variable.

## Results

**Fig. 1:**
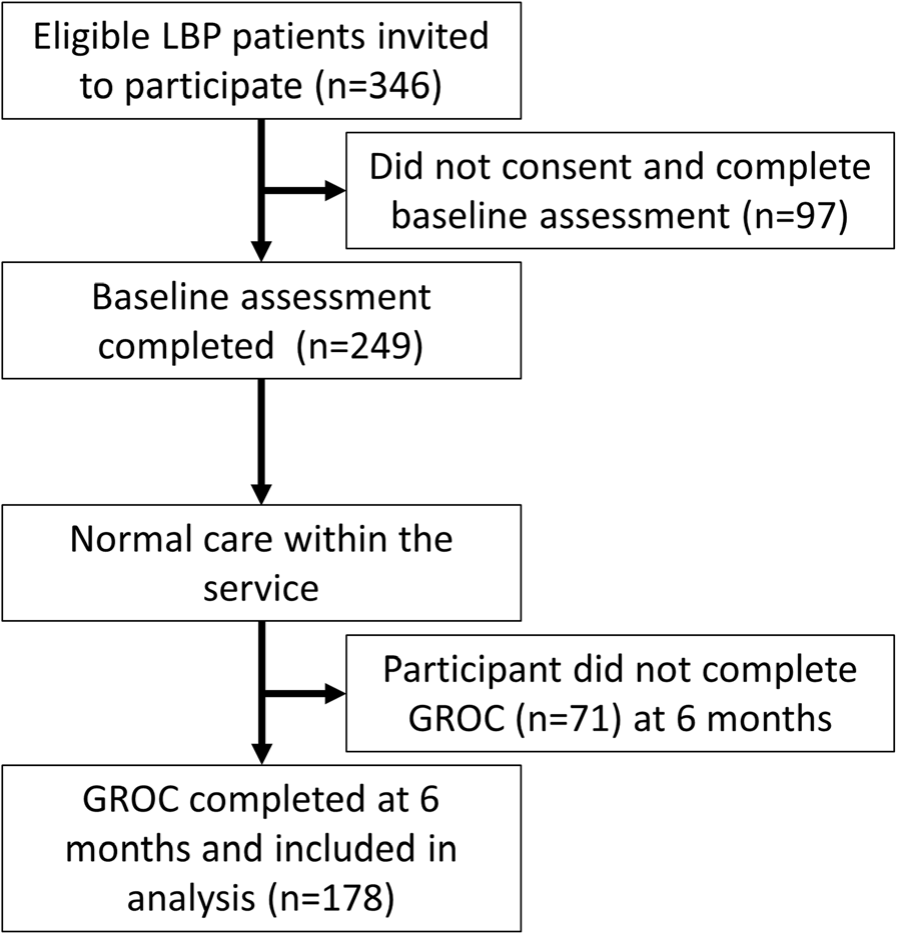
Flow diagram of the study

The flow of participants through the study is depicted in Fig. 1. A total of 346 patients were initially deemed eligible following screening and invited to participate. Of these 249 participants completed written consent forms and baselines measures. At the 6 month follow up period GROC measures were received from 178 participants, representing a 28.5% drop out rate.

For the 178 patients who completed the 6 month follow up GROC measure, the median (IQR) GROC score was + 2 (0 to + 4) with the range of scores from − 6 to + 7. Overall, 79 of the 178 (44.4%) patients who had completed the 6 month follow up GROC measure fitted the criteria as a non-responder.

The characteristics of participants classified as non-responders or responders is described in Table 1.

Ten potential explanatory variables demonstrated a significant univariate relationship (*p* ≤ 0.1) with a poor response to non-surgical management of LBP as shown in Table 1.

Of the 10 variables, 7 were retained in the final multivariable model. Specifically, the QOL, PSEQ and ÖMPSQ measures were found to be strongly correlated with the ODI (r_s_ = 0.6–0.7, *p* < 0.001). The ODI was retained based on likely clinical relevance as a widely used measure of pain related disability in LBP disorders (investigators’ judgement). In addition, when these excluded variables (PSEQ, QOL, ÖMPSQ) were substituted into the multivariable as sensitivity analyses, findings were generally consistent with the final model although with a poorer model fit [40] (not presented).

Variables retained in the final model observed to be predictive of a poor response to the non-surgical management of LBP are shown in Table 2. These variables included; 1/ lower patient Formal Education Level with the OR (2.67 (95%CI 1.17–6.09), *p* = 0.02) indicating that participants who did not complete school (School Incomplete) were at greater odds of a poor response than those who ‘Completed TAFE/Trade/ University’; and 2/ higher self-reported back disability (ODI) with the OR 1.33 (95%CI 1.01–1.77) per 10/100 point score increase, *p* = 0.046) indicating the odds of a poor response were higher among those reporting higher back disability on this index. The service site was not a significant factor in the model. Exploratory tests (independent t-test and chi-square) suggested that those who completed their outcome measures at follow-up were not different to those who did not on the basis of the significant variables of back disability level (ODI, *p* = 0.27) and level of highest education (*p* = 0.15).

**Table 2:** Final model showing patient characteristics demonstrating a relationship with reporting a poor response (GROC ≤ + 1) to the non-surgical multidisciplinary management of LBP. Specifically, not having completed school (compared to having completed Tafe/Trade/University) and a higher Oswestry Disability Index (ODI) score significantly increased the odds of a poor response (OR > 1)

Variables	Adjusted OR (95% Confidence Intervals)	***P***- Value
**Demographic and Social Measures**		
Gender (male)	1.53 (0.69–3.36)	0.29
Highest Level of Education		
Completed Tafe/Trade/ University (yes)	Referent	
Completed Secondary School (yes)	2.16 (0.73–6.38)	0.16
School Incomplete (yes)	2.67 (1.17–6.09)	0.02
Work Status		
Employed (yes)	Referent	
Unemployed (yes)	1.34 (0.42–4.27)	0.62
Retired (yes)	1.03 (0.44–2.4)	0.94
**General and Global Health Measures**		
Disability Benefits (yes)	1.52 (0.49–4.73)	0.47
Smoking Status (yes)	1.37 (0.57–3.28)	0.48
**Condition Specific Symptoms and Signs**		
ODI (per 10/100 points increase in score)	1.33 (1.01–1.77)	0.046
Symptom Duration (yes, > 12 months)	2.09 (0.79–5.53)	0.14

*GROC* Global Rating of Change, model C-Statistic (95%) = 0.72 (0.64–0.81)

## Discussion

Patient characteristics increasing the odds of a poor response to non-surgical multidisciplinary management of LBP were explored within an advanced practice physiotherapist-led multidisciplinary orthopaedic tertiary care service. Those reporting a more severely disabling LBP condition and a low formal level of education (school not completed) at their initial service consultation were at greater odds of not responding favourably. However the relative width and approximation to 1 of the lower boundary of the odds ratios for these characteristics (see Table 2) indicate their impact on outcome has a range of uncertainty. Therefore the presence of these characteristics in individuals with LBP does not negate their participation in a trial of this form of management. Instead these patients may require a more targeted supported approach to management and more diligent monitoring of outcomes to ensure timely referral to an alternative care pathway if progress is inadequate.

Lower levels of education have been strongly linked to poor health outcomes [41–43] including a poorer response to interventions for LBP [11, 44, 45]. While the link between a lower level of education and poorer response to pragmatic, multidisciplinary, non-surgical management observed in this study is most likely complex, it may relate to the patient’s level of health literacy [46, 47]. Health literacy refers to an individual’s capacity to seek, understand, and use health information [48]. Health literacy has been reported as a potential mechanism explaining the relationship between a low level of education and poor health [46]. Poorer health literacy is reported to negatively influence disability, and present barriers to positive health behaviours in some with chronic LBP [47, 49]. However it should be acknowledged that no formal measure of health literacy was evaluated in this study. Therefore this inferred relationship between low education level and low health literacy although supported by the literature [46] is speculative in this study.

The observed relationship between a higher initial reported back disability level and poor response to management is testament to the need for studies to be specific to LBP patient populations, healthcare settings, and interventions. These findings are in contrast to previous studies investigating treatment response to unimodal interventions (acupuncture, manipulation) for LBP in primary care, identifying those with higher disability being more likely to achieve a better response [11, 44, 50, 51]. While a greater level of pain related disability at the initial consultation may represent greater numerical potential for improvement, a severe level of self-reported disability as evident in the poor responders group (average ODI score of 46.1%) in this study may reflect a certain threshold of severity beyond which attaining benefit in response to multidisciplinary non-surgical management is more challenging. This notion is supported in this sample of patients in Table 1 showing potential explanatory variables with a univariate relationship with outcome. Here higher self-reported back disability was strongly related (r_s_ = 0.6–0.7) to more severe psychological (lower PSEQ score, higher ÖMPSQ score) and general/global health (lower QOL score) features (not included in the final model due to collinearity issues). Collectively this may reflect a more severe and potentially more multifactorial LBP condition being more challenging to positively benefit, even in response to a multidisciplinary program designed to pragmatically address the mix of biopsychosocial factors potentially underlying a patient’s LBP presentation [3, 4].

There were consistencies and inconsistencies in the findings with previous literature. In contrast to our previous study in the same health service [6] the presence of coexisting cervical and/or thorax pain was not associated with a poor response. Similarly patient expectations concerning treatment benefit was not related to a poorer outcome in contrast to previous studies in LBP and other chronic pain conditions [52, 53]. Consistent with previous literature was an absence of relationship between patient reported outcome and physical measures [54]. Similarly, there was no apparent association between radiological findings and outcome consistent with previous literature in LBP [55–58]. Potentially inconsistencies in findings between studies may reflect service and/or methodological differences between studies. More likely though discrepancies between studies attests to the multidimensional nature of LBP [58], supported by the observed low numbers, and relatively modest association coefficients, of explanatory variables showing a relationship with outcome in this study. Overall it would appear that marked variability of LBP presentations seen within these tertiary care settings presents challenges in accurately identifying those at risk of a poor response.

The findings have both clinical and future research implications. Reflecting on the significant impact of education level on outcome, potentially critical inroads to effective patient care may be being missed by inadequate consideration of health literacy. It is estimated that 60% of adult Australians may have low individual health literacy skills [59] which is a similar metric to other countries [60]. Some patients with a lower level of formal education may require additional self-management support initiatives to improve their capacity to understand and use health information [49]. In fact recent literature recommends clinicians self-evaluate health literacy strategies within their service with a focus on how information is presented to patients [61]. Suggested strategies to facilitate health literacy include simplifying the focus of consultations and provided information, avoiding medical jargon, and assessing patient comprehension of information [60, 62]. While these may be useful strategies, health literacy is a complex topic beyond the scope of this paper and is addressed more comprehensively in other literature [60, 63]. Health literacy was also not measured specifically in this study. Future studies will need to specifically evaluate the relationship between health literacy skills and outcome within this service, as well as evaluate the impact of implemented health literacy enhancement strategies [61] on outcomes within the service.

### Limitations

Sample size was a limitation of this study potentially impacting findings. While participants completing baseline assessments fell only marginally short of the projected required sample size (249/264) (funding limitations prevented further recruitment), loss to follow-up was larger than anticipated (28.5%). In this context, it is important to consider the odds ratios and confidence intervals in the context of this sample size limitation. For example, despite the inability to detect a statistically significant association in the final model in this sample for the symptom duration of greater than 12 months (*p* = 0.14) variable, the odds ratio point estimate (OR = 2.09) and 95% confidence intervals (0.79–5.53) indicate it was possible that findings of this nature could be a false negative and further investigation (or meta-analyses) are perhaps warranted in this context. It is also possible that differences in LBP characteristics and management response may have existed between participants who did and did not provide the 6 month outcome measures, however they did not differ at baseline in the variables shown to be significant in the final model (ie. disability level (ODI) or level of highest education (*p* > 0.14)). Findings are also most directly relevant to patients with LBP managed within these tertiary care settings and may not directly apply to other LBP populations managed, for example, in primary care. The patients in this study managed in tertiary care are potentially typically older (average (SD) age 53.69 ± 15.31) years) and more severely disabled (average (SD) ODI 41.13 ± 15.93 points/100) than the general LBP population who may have different characteristics affecting management outcomes. Findings may also be specific to the patient characteristics recorded and outcome used (ie GROC measure) which may have limitations such as recall bias [64].

Findings also may only be relevant to the nature of the multidisciplinary service included in this study. Varied characteristics of different multidisciplinary programs such as contact time, program philosophy, nature and intensity of program components, and experience and skill of involved clinicians are considered to impact a programs efficacy [3]. The potential clinical meaningfulness of the study findings is however strengthened by collection of data across multiple sites. Measures used were also simple and could be standardly applied between sites and clinicians, enhanced by prior training. With the number of clinicians collecting measures across sites however, we are unable to be sure that some variation in measurements existed contributing to potential measurement error.

## Conclusions

Patients presenting with LBP to advanced practice physiotherapist-led multidisciplinary orthopaedic services in tertiary care may be at greater odds of a poor response to non-surgical multidisciplinary management if they have a low level of formal education, and at the initial consultation report high levels of pain related back disability. Potentially patients with these characteristics may need a more supported approach to management such as an evaluation and enhancement of their health literacy skills, though this needs further investigation within this service and patient population. Clinical outcomes in patients with these characteristics may also need to be more closely monitored so as to avoid unproductive management. While the findings of this study may be of interest to clinicians managing LBP in the general population, findings are most likely directly relevant to patients managed in comparable tertiary care settings.

## Data Availability

The dataset from this study is not publicly available due to the data having been collated from multiple hospital health services each with individual data custodians that require further approval for access. Please contact corresponding author (s.oleary@uq.edu.au) regarding any data requests.

## Abbreviations

LBP: Low Back Pain
OR: Odds Ratio
ODI: Oswestry Disability Index
GROC: Global Rating of Change
